# Pre-analytical storage effects on ALU- and LINE1-derived cell-free DNA biomarkers in whole blood and plasma

**DOI:** 10.17305/bb.2026.13409

**Published:** 2026-01-07

**Authors:** Lifang Zhao, Chao Ying, Songnian Hu, Xuemin Wang, Qimeng Li, Yanning Cai

**Affiliations:** 1 Department of Central Laboratory and Clinical Biobank, Xuanwu Hospital, Capital Medical University, Beijing, China; 2 Beijing Geriatric Medical Research Center, Beijing, China; 3 Department of Neurobiology, Xuanwu Hospital, Capital Medical University, Beijing, China; 4 Key Laboratory of Neurodegenerative Diseases, Ministry of Education, Beijing, China; 5 School of Rehabilitation Medicine, Gannan Medical University, Ganzhou, Jiangxi, China

**Keywords:** Cell-free DNA, Arthrobacter luteus repeats, long interspersed nuclear elements 1, biomarkers, pre-analytical factors

## Abstract

Cell-free DNA (cfDNA) biomarkers derived from *Arthrobacter luteus* (ALU) repeats and long interspersed nuclear elements 1 (LINE1) — including ALU-115, ALU-247, LINE1-97, and LINE1-266 concentrations, as well as the integrity ratios ALU-247/115 and LINE1-266/97 — are commonly utilized to assess cfDNA quantity and integrity. This study examined the impact of delayed blood processing and prolonged plasma storage on these biomarkers using quantitative polymerase chain reaction. Blood samples were collected from twelve healthy individuals (6 males; mean age, 65.8 ± 4.69 years) into dipotassium ethylenediaminetetraacetic acid tubes. Plasma cfDNA was extracted after various storage durations and temperatures, with aliquots from immediately processed blood subsequently stored at −80 ^∘^C for different time intervals. Except for LINE1-97, most biomarkers showed significantly higher levels in plasma isolated from whole blood stored at room temperature compared to plasma processed immediately. Storage at 4 ^∘^C resulted in fragment-specific effects: ALU-247/115 levels remained stable at 3 hours but decreased at 6 hours, while LINE1-266/97 levels increased at both time points. For plasma stored at −80 ^∘^C, ALU-derived biomarkers remained stable for up to 12 months; however, LINE1-97 levels significantly declined, accompanied by a corresponding increase in LINE1-266/97 as early as one month after freezing. These findings indicate that both storage duration and temperature significantly impact the measured levels of ALU- and LINE1-derived cfDNA biomarkers. Consequently, standardization of pre-analytical handling of blood and plasma is crucial for studies evaluating cfDNA quantity and integrity.

## Introduction

Circulating cell-free DNA (cfDNA) consists of fragmented DNA molecules that are present in bodily fluids, particularly plasma, outside of cells. These fragments primarily originate from processes such as apoptosis, necrosis, NETosis, and active secretion [1–3]. Apoptotic cells typically release DNA fragments measuring 180–200 base pairs (bp), while tumor necrosis results in fragments of variable lengths, generally exceeding 200 bp [2, 3]. Elevated levels of longer DNA fragments in circulation have been recognized as significant indicators of tumor-derived DNA [4, 5]. A crucial parameter reflecting cfDNA fragmentation is the cfDNA integrity (cfDI) index, which is calculated as the concentration ratio of longer to shorter fragments at the same genetic locus. Due to its easy accessibility from peripheral blood, cfDNA serves as a promising biomarker for disease diagnosis, prognosis, and therapeutic monitoring [5–8].

Despite the increasing interest in cfDNA applications, clinical translation is hindered by the absence of standardized pre-analytical procedures [9, 10]. The quality and yield of cfDNA are influenced by multiple pre-analytical steps, from sample collection to analysis [10]. Plasma is preferred over serum for cfDNA isolation, as serum is more susceptible to contamination with genomic DNA (gDNA) released from leukocytes during clotting [11, 12]. Ethylenediaminetetraacetic acid (EDTA) effectively inhibits deoxyribonuclease (DNase) activity, making EDTA-coated tubes the most commonly used for cfDNA analysis [13, 14]. Double centrifugation at either 4 ^∘^C or room temperature (RT) is generally recommended to minimize gDNA contamination [15, 16]. Numerous studies have evaluated the effects of delayed blood processing on cfDNA measurements; however, permissible delay times exhibit considerable variability [11, 12, 16–24]. Furthermore, optimal plasma storage conditions prior to cfDNA extraction remain inadequately defined, with previous studies reporting inconsistent findings regarding the impact of storage at −80 ^∘^C on cfDNA concentrations [11, 17, 25].

Short interspersed nuclear elements (SINEs) and long interspersed nuclear elements (LINEs) are abundant and well-characterized repetitive sequences within the human genome. The *Arthrobacter luteus* (ALU) repeat, named after the restriction endonuclease isolated from the bacterium *Arthrobacter luteus*, is the most prevalent SINE, constituting at least 11% of the genome [26]. Long interspersed nuclear element 1 (LINE1) sequences, which comprise approximately 17% of the genome, represent the largest retrotransposon family [27]. Biomarkers derived from ALU and LINE1 sequences are extensively utilized in cancer diagnosis, prognosis, and monitoring [7, 28–34]. The concentrations of shorter fragments, ALU-115 and LINE1-97, reflect total cfDNA levels, while the concentrations of longer fragments, ALU-247 and LINE1-266, are considered indicators of non-apoptotic cfDNA. The integrity ratios ALU-247/115 and LINE1-266/97 are commonly employed to assess cfDI [35, 36].

For cfDNA-based analyses to achieve clinical applicability, measurement reproducibility must be ensured. While several studies have investigated how pre-analytical factors influence the concentration of specific cfDNA fragments in plasma [11, 13, 17, 18, 21], direct comparisons of conditions affecting ALU- and LINE1-derived biomarkers remain limited. To address this gap, we independently examined the effects of delayed plasma preparation from whole blood stored at RT and at 4 ^∘^C, along with the impact of long-term plasma storage at −80 ^∘^C, on levels of these widely used cfDNA biomarkers.

## Materials and methods

### Study subjects

Twelve healthy Han Chinese volunteers (6 males and 6 females; mean age 65.8 ± 4.69 years) from Xuanwu Hospital, Capital Medical University, participated in this study.

### Sample collection and cfDNA extraction

Samples were collected during two independent blood donation events: the first in May 2024 and the second in March 2025. Due to insufficient plasma volume from one male participant, only 11 participants provided complete samples for the initial experiment assessing cfDNA stability under prolonged whole-blood and plasma storage (6 females and 5 males; mean age 64.5 ± 5.72 years). For the subsequent experiment involving 4 ^∘^C whole-blood storage, sufficient plasma was available from all participants, allowing for the inclusion of the full cohort (*n* ═ 12).

**Figure 1.**
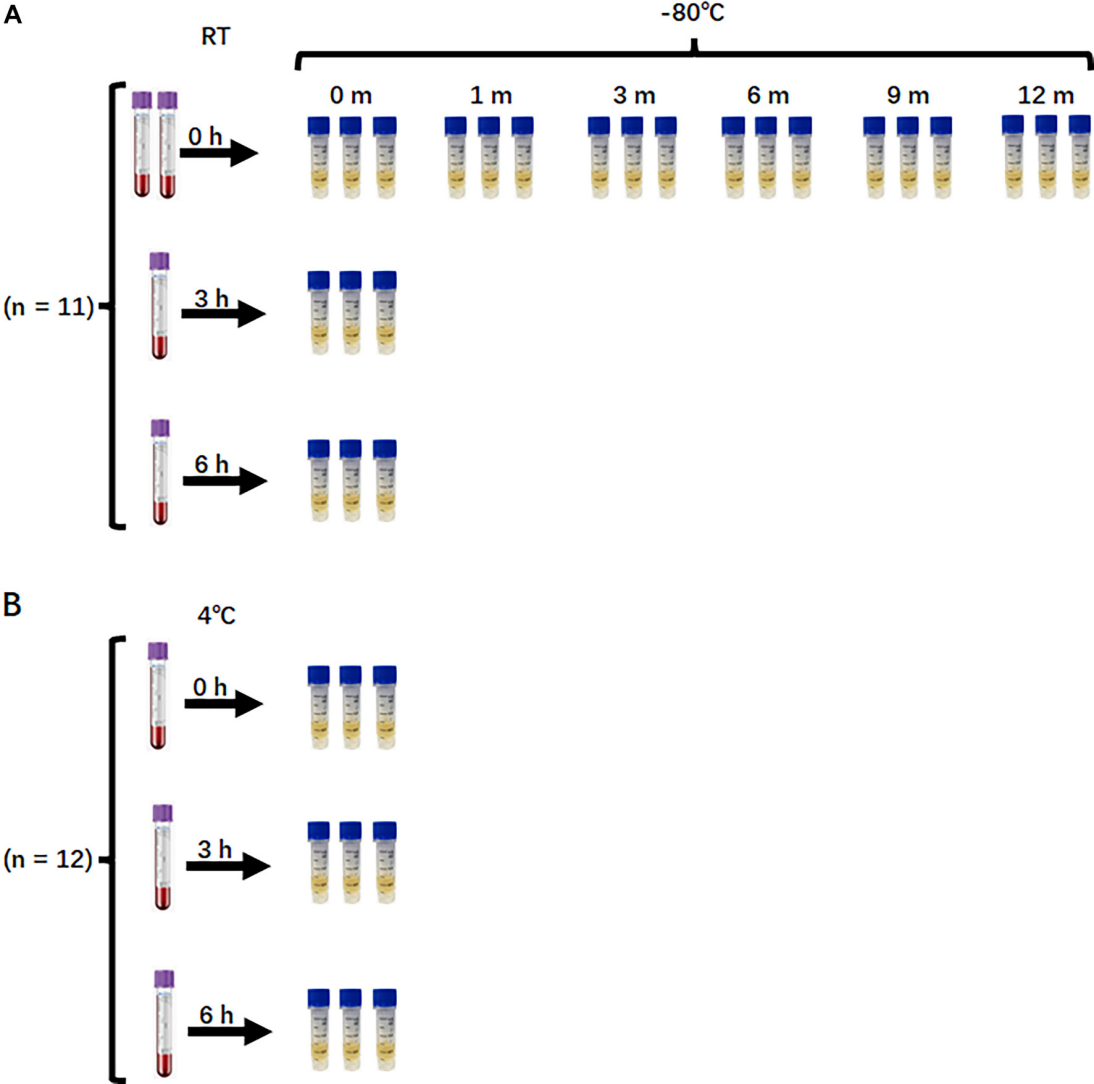
**Experimental design.** (A) RT whole-blood holding and long-term plasma storage experiment (*n* ═ 11). Venous blood was collected into four dipotassium EDTA tubes (two 10-mL and two 4-mL). Blood from the two 10-mL tubes was processed immediately (0 h) by double centrifugation; plasma was aliquoted (500 µL; three aliquots per time point) and stored at −80 ^∘^C for 0, 1, 3, 6, 9, or 12 months before cfDNA extraction. The remaining two 4-mL tubes were held at RT for 3 or 6 h, then processed identically; resulting plasma aliquots were stored at −80 ^∘^C and extracted within 3 days. (B) Refrigerated whole-blood holding experiment (*n* ═ 12). Venous blood was collected into three 4-mL dipotassium EDTA tubes and stored at 4 ^∘^C for 0, 3, or 6 h prior to double centrifugation. Plasma was aliquoted (500 µL; three aliquots per time point), stored at −80 ^∘^C, and used for cfDNA extraction within 3 days. Abbreviation: RT: Room temperature.

In the first experiment, fasting venous blood samples from 11 participants were collected into two 10-mL and two 4-mL dipotassium EDTA-coated plastic tubes (Becton, Dickinson and Company, Franklin Lakes, NJ, USA; Cat# 366643 and 367863) for plasma preparation. Samples from the two 10-mL tubes were processed immediately by centrifugation at 1600 *×g* for 10 min at 4 ^∘^C. The supernatant was carefully transferred and centrifuged again at 16,000 *×g* for 10 min at 4 ^∘^C. The resulting 10 mL of plasma from each participant was aliquoted into 18 individual 500 µL tubes, with three aliquots designated for cfDNA extraction at each storage time point, and stored at −80 ^∘^C for analysis after 0, 1, 3, 6, 9, or 12 months. Samples from the two 4-mL tubes were stored at RT for 3 or 6 h prior to centrifugation under the same conditions. The plasma aliquots prepared for the RT delay experiment were stored at −80 ^∘^C and subjected to cfDNA purification within 3 days (Figure 1A).

In the second experiment, fasting venous blood from 12 participants was collected into three 4-mL EDTA-coated tubes and stored at 4 ^∘^C for 0, 3, or 6 h before centrifugation under the same conditions as in the first experiment (Figure 1B). The isolated plasma was aliquoted, stored at −80 ^∘^C, and used for cfDNA isolation within 3 days.

For each participant and each time point, three independent plasma aliquots (500 µL each) were prepared, stored, and subsequently utilized for cfDNA extraction. cfDNA was isolated from 500 µL plasma aliquots using a single freeze-thaw cycle with the FineMag Plasma Cell-Free DNA Extraction Kit (GENFINE Biotech; Changzhou, China; Cat# M107ST) according to the manufacturer’s instructions. The cfDNA was eluted in 65 µL of elution buffer and stored at −80 ^∘^C until analysis.

### Quantification of cfDNA concentration and integrity

The concentration and integrity of cfDNA were quantified using quantitative polymerase chain reaction (qPCR) targeting two repetitive elements: ALU and LINE1. For each target, both short fragments (ALU-115 bp; LINE1-97 bp) and long fragments (ALU-247 bp; LINE1-266 bp) were amplified in triplicate with the LightCycler 480 SYBR Green I Master Mix (Roche, Mannheim, Germany; Cat# 04887352001) on the Roche LightCycler^®^ 480 system (Roche, Mannheim, Germany; Cat# 05015278001). Primers were selected from previously published studies [35, 36] and are detailed in Table S1.

Each 20 µL qPCR reaction included 2 µL of cfDNA template, 0.4 µL of forward and reverse primers (10 µM), 10 µL of 2× SYBR Green master mix, and 7.2 µL of nuclease-free water. The reaction conditions consisted of an initial denaturation at 95 ^∘^C for 15 s, followed by 35 cycles of denaturation at 95 ^∘^C for 15 s, annealing at 65 ^∘^C or 60 ^∘^C for 20 s, and extension at 72 ^∘^C for 30 s. Short amplicons were nested within the corresponding long amplicons, and expected product sizes were verified using 2% agarose gel electrophoresis (Figure S1). Calibration curves were constructed using ten-fold serial dilutions of purified gDNA extracted from peripheral blood leukocytes of a healthy volunteer, following the QIAamp DNA Blood Mini Kit protocol (Qiagen, Hilden, Germany; Cat# 51104). The concentration and purity of the extracted gDNA were measured with a NanoDrop 2000spectrophotometer (Thermo Fisher Scientific, Waltham, MA, USA). Only gDNA samples with an A_2__6__0_/A_2__8__0_ ratio between 1.8 and 2.0, indicating high purity and minimal protein or phenol contamination, were utilized for serial dilutions and calibration curve construction, starting at 10 ng/µL and diluting to 0.1 pg/µL. All primer sets maintained linearity across six orders of magnitude, with logarithmic regression lines yielding R^2^ values greater than 0.99. The detection limit reached 0.01 pg, and amplification efficiency for all primer sets ranged from 91.7% to 100.8% (Table S1). Melting curve analyses confirmed the specificity of each assay, with a single peak observed for each reaction. Water served as the no-template control on each reaction plate. To account for inter-plate variability, a control DNA sample was included on every plate. The concentration measured for each control sample was divided by the overall mean concentration of that control across all plates to generate a normalization factor. Mean concentrations for each fragment were calculated from triplicate reactions. If the coefficient of variation among the triplicates exceeded 15%, the sample was re-analyzed until the variability fell below this threshold. Absolute quantification was performed using the LightCycler^®^ 480 software, and cfDI was calculated as the ratio of long to short fragment concentrations. qPCR triplicate measurements were averaged for each extraction, and these values were subsequently averaged across three independent plasma extractions to generate a single per-participant value at each time point for statistical analysis.

### Ethical statement

The study protocol received approval from the Xuanwu Hospital Medical Research Ethics Committee and Institutional Review Board (approval No. [2024]045; April 29, 2024) and was conducted in accordance with the *Declaration of Helsinki*. All study participants provided written informed consent prior to enrollment.

### Statistical analysis

The sample size was not predetermined using statistical methods; however, it was comparable to sample sizes reported in previous studies in this field [13, 16–19, 21, 23, 24]. Statistical analyses were performed using the Statistical Package for the Social Sciences (IBM SPSS Statistics for Windows, Version 27.0; IBM Corp., Armonk, NY, USA). Graphs were generated using GraphPad Prism version 9.0.0 (GraphPad Software, Boston, MA, USA; www.graphpad.com). Data normality was assessed using the Shapiro-Wilk test, skewness and kurtosis statistics, and visual inspection of Q-Q plots. Homogeneity of variances was evaluated using Levene’s test. Normally distributed data are presented as mean ± standard deviation (SD) and were compared using repeated-measures analysis of variance (ANOVA) to examine differences in cfDNA concentration and cfDI across storage conditions. Skewed data are reported as median and interquartile range (IQR) and were analyzed using the Friedman test. When repeated-measures ANOVA indicated a significant effect, post-hoc pairwise comparisons were performed to identify differences between measurement time points or experimental conditions. If the assumption of sphericity was met, paired *t*-tests with Bonferroni correction were utilized. If Mauchly’s test indicated a violation of sphericity, Greenhouse-Geisser-adjusted degrees of freedom were applied in the ANOVA, followed by Bonferroni-adjusted post hoc comparisons. For non-normally distributed repeated-measures data analyzed with the Friedman test, post-hoc comparisons were conducted using the Wilcoxon signed-rank test with Bonferroni correction. Adjusted *p* values are reported for all multiple comparisons, and a two-tailed *p* < 0.05 was considered statistically significant.

## Results

### Effect of blood storage at RT on cfDNA concentration and integrity

**Figure 2.**
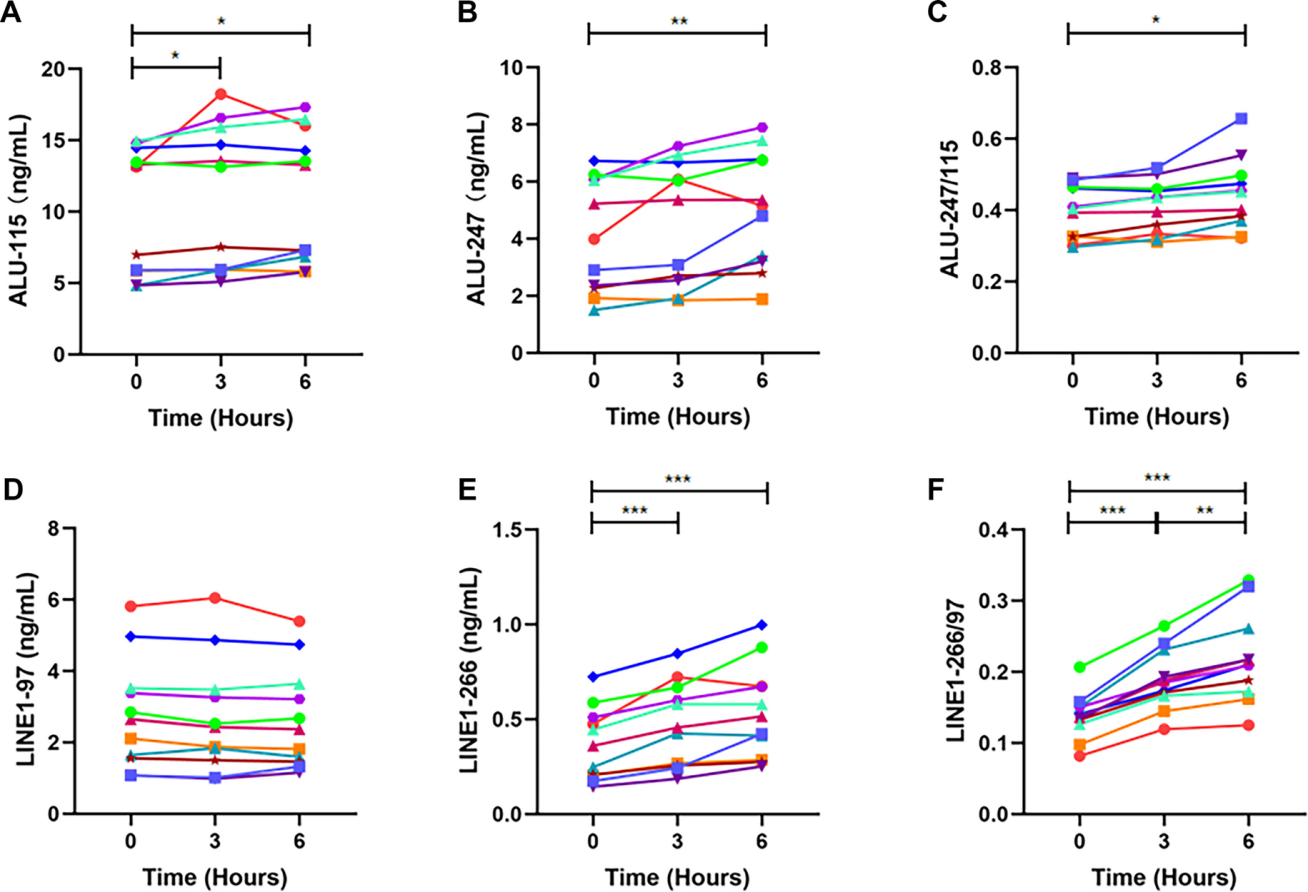
**Plasma levels of ALU- and LINE1-derived biomarkers following delayed blood processing at RT.** This figure illustrates the levels of ALU-115 (A), ALU-247 (B), ALU-247/115 (C), LINE1-97 (D), LINE1-266 (E), and LINE1-266/97 (F) in plasma samples from blood stored at room temperature and processed at various time intervals post-collection. Individual specimen measurements are represented by different colors. Differences among time points were evaluated using a repeated-measures analysis (ANOVA or Friedman test, as appropriate). When a significant overall time effect was identified, post-hoc pairwise comparisons were performed using paired *t* tests or Wilcoxon signed-rank tests to determine differences between storage time points, with Bonferroni correction applied for multiple comparisons (*n* ═ 11). *p < 0.05, **p < 0.01, ***p < 0.001. Abbreviations: ALU: Arthrobacter luteus; LINE1: Long interspersed nuclear element 1; RT: Room temperature; ANOVA: Analysis of variance.

To evaluate the impact of RT storage on ALU- and LINE1-derived cfDNA biomarkers, blood samples were stored at RT for 0, 3, or 6 h prior to plasma preparation. Plasma cfDNA was then extracted and analyzed via qPCR (Figure 2, Table S2).

Compared to plasma processed immediately after venipuncture (0 h), ALU-115 fragment concentrations were significantly higher after 3 and 6 h of RT storage (*p* ═ 0.042 and 0.019, respectively). In contrast, ALU-247 concentrations were comparable after 3 h (*p* ═ 0.137) but significantly increased after 6 h (*p* ═ 0.007). No significant difference in ALU fragment levels was observed between the 3-h and 6-h storage periods (ALU-115: *p* ═ 0.365; ALU-247: *p* ═ 0.184; Figure 2A and 2B). LINE1-97 concentrations remained stable across all RT storage time points, whereas LINE1-266 levels increased markedly after both 3 and 6 h of RT storage (both *p* < 0.001, Figure 2D and 2E). Analysis of cfDI revealed a significant increase in the ALU-247/115 ratio after 6 h of RT storage compared to 0 h (*p* ═ 0.021). Similarly, LINE1-266/97 ratios were significantly elevated after both 3 and 6 h relative to 0 h (both *p* < 0.001), and cfDI values were significantly higher at 6 h than at 3 h for LINE1-266/97 (*p* ═ 0.004, Figure 2C and 2F).

These findings suggest that prolonged RT storage facilitates the release of background gDNA from lysed leukocytes, resulting in elevated cfDNA fragment concentrations and increased cfDI values [11, 12, 37]. However, the extent of these effects varies among different cfDNA species.

### Effect of blood storage at 4 ^∘^C on cfDNA concentration and integrity

**Figure 3.**
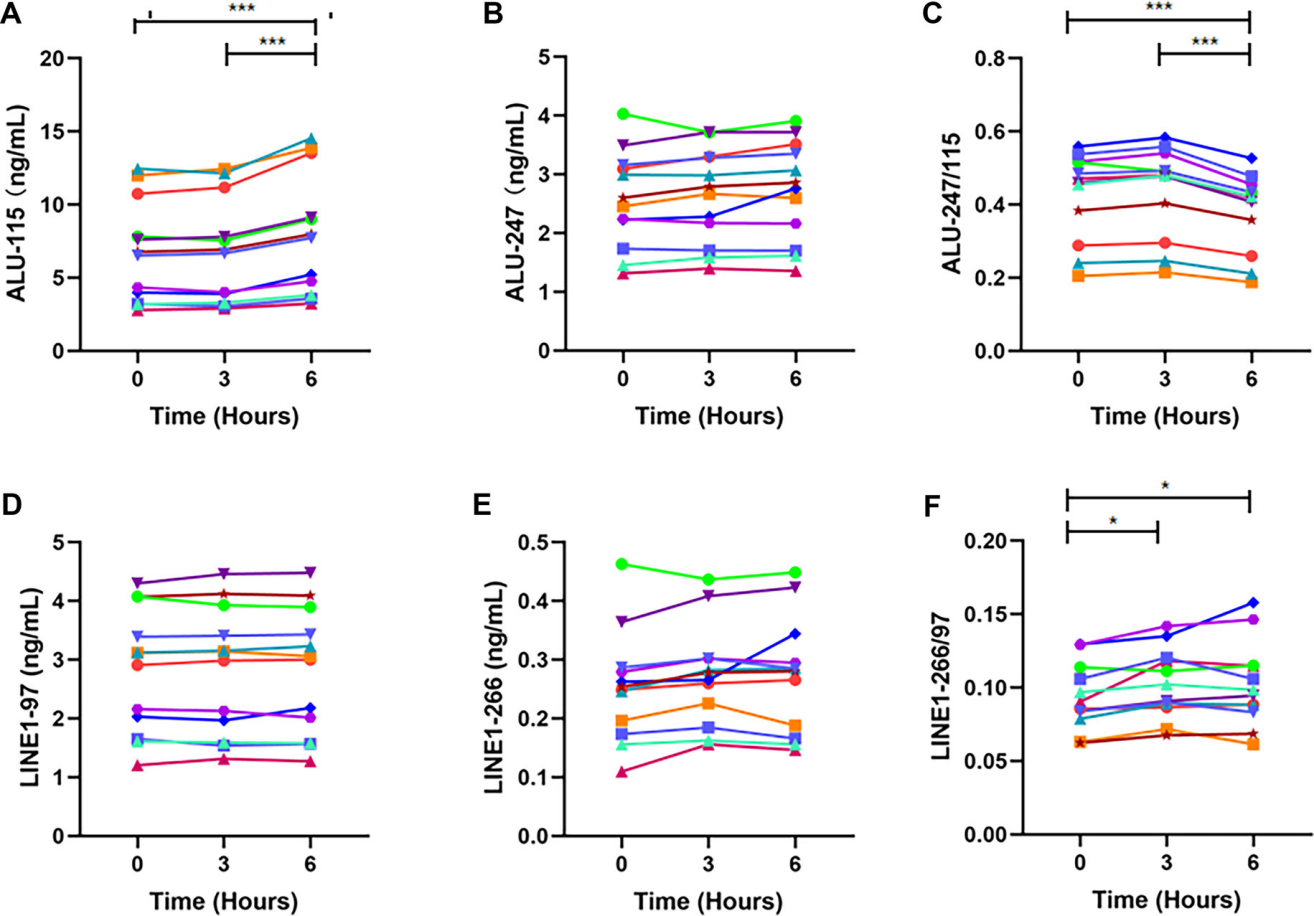
**Plasma levels of ALU- and LINE1-derived biomarkers following delayed blood processing at 4 ^∘^C.** This figure illustrates the levels of ALU-115 (A), ALU-247 (B), ALU-247/115 (C), LINE1-97 (D), LINE1-266 (E), and LINE1-266/97 (F) measured in plasma from blood samples stored at 4 ^∘^C and processed at various time intervals post-collection. Measurements from individual specimens are represented in distinct colors. Differences among time points were evaluated using a repeated-measures framework (ANOVA or Friedman test, as appropriate). When a significant overall time effect was observed, post-hoc pairwise comparisons were performed using paired *t* tests or Wilcoxon signed-rank tests to determine differences between storage time points, with Bonferroni correction applied for multiple comparisons (*n* ═ 12). *p < 0.05, **p < 0.01, ***p < 0.001. Abbreviations: ALU: Arthrobacter luteus; LINE1: Long interspersed nuclear element 1; ANOVA: Analysis of variance.

This study evaluates the impact of refrigerated storage on cfDNA biomarkers by storing blood samples at 4 ^∘^C for 0, 3, or 6 h prior to plasma separation. The cfDNA in the plasma was then purified and analyzed using qPCR (Figure 3, Table S3).

At 4 ^∘^C, ALU-247 levels remained stable for the entire 6-h storage period, while ALU-115 levels were stable only for the first 3 h. ALU-115 concentrations increased significantly at 6 h compared to both immediate processing and the 3-h storage mark (both *p* < 0.001, Figure 3A and 3B). Consequently, the ALU-247/115 ratio was significantly lower at 6 h than at 0 or 3 h (both *p* < 0.001, Figure 3C). In contrast, LINE1-97 and LINE1-266 levels remained unaffected by 4 ^∘^C storage for up to 6 h (Figure 3D and 3E). However, the LINE1-266/97 ratio increased significantly after both 3 h and 6 h of storage (*p* ═ 0.010 and 0.047, respectively), with no significant difference between these two time points (Figure 3F).

These results indicate that ALU- and LINE1-derived cfDNA biomarkers exhibit distinct response patterns to short-term refrigerated storage, with some fragment ratios decreasing and others increasing over time. The potential underlying mechanisms are discussed in detail in the Discussion section.

### Effect of plasma storage at −80 ^∘^C on cfDNA concentration and integrity

**Figure 4.**
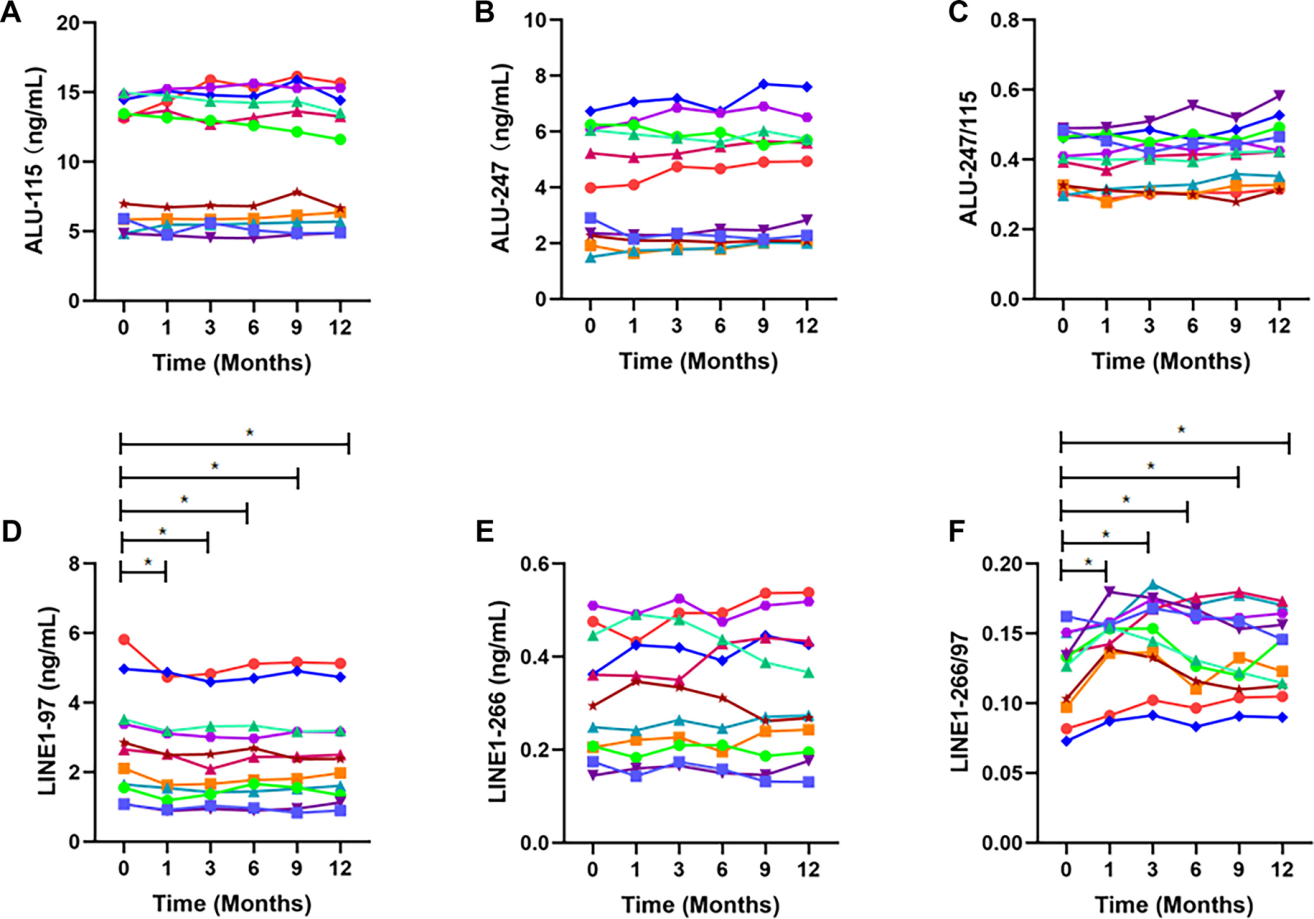
**Long-term stability of ALU- and LINE1-derived biomarkers in plasma stored at −80 ^∘^C.** This figure presents the levels of ALU-115 (A), ALU-247 (B), ALU-247/115 (C), LINE1-97 (D), LINE1-266 (E), and LINE1-266/97 (F) in plasma samples stored at −80 ^∘^C for varying durations. Measurements from individual specimens are represented in distinct colors. Differences among time points were analyzed using a repeated-measures framework, employing ANOVA or the Friedman test as appropriate. When a significant overall time effect was identified, post-hoc pairwise comparisons were performed using paired *t* tests or Wilcoxon signed-rank tests to determine differences between storage time points, with Bonferroni correction applied for multiple comparisons (*n* ═ 11). *p < 0.05. Abbreviations: ALU: Arthrobacter luteus; LINE1: Long interspersed nuclear element 1; ANOVA: Analysis of variance.

To assess the stability of cfDNA during long-term storage, plasma aliquots from fresh blood samples were stored at −80 ^∘^C for up to 12 months before cfDNA extraction and analysis (Figure 4, Table S4).

Plasma ALU-115 and ALU-247 concentrations remained stable throughout the 12-month storage period (Figure 4A and 4B). Consistent with these findings, the ALU-247/115 ratios showed no significant changes at any of the tested storage time points when compared with fresh plasma (all *P* > 0.05, Figure 4C). In contrast, LINE1-97 concentrations declined significantly as early as one month post-freezing (*p* ═ 0.036) and remained significantly lower at all subsequent time points (all *p* < 0.05, Figure 4D). LINE1-266 levels remained unchanged throughout the storage period (all *p* > 0.05, Figure 4E). Consequently, the LINE1-266/97 ratio increased significantly within the first month and remained elevated thereafter (all *p* < 0.05, Figure 4F).

These findings demonstrate that different cfDNA fragments exhibit variable stability during long-term frozen storage, with LINE1-97 being particularly susceptible to degradation. Possible mechanisms are described in the Discussion.

## Discussion

Circulating biomarkers in peripheral blood, including cfDNA, possess considerable clinical potential. However, standardized guidelines for blood handling to ensure reliable cfDNA analysis remain inadequate. In this study, we systematically evaluated the effects of delayed blood processing and prolonged plasma storage on commonly used ALU- and LINE1-derived cfDNA biomarkers. By assessing samples stored at RT and 4 ^∘^C for various durations, as well as plasma stored at −80 ^∘^C over multiple time points, we demonstrate that storage conditions and duration can significantly influence cfDNA measurements.

Previous studies have reported considerable variability regarding the permissible delay before plasma separation in cfDNA research. Some studies suggest cfDNA remains stable for up to 4 h at RT or 24 h at 4 ^∘^C when collected in EDTA tubes [19, 22–24]. However, these studies generally quantified total cfDNA concentration using fluorometric assays, which may not accurately reflect the stability of specific cfDNA fragments. When examining individual gene fragments, Jung et al. and Lam et al. reported no significant changes in cfDNA levels, measured using a 110-bp β-globin fragment, after blood storage at RT for up to 8 h and 6 h, respectively [13, 17]. Similarly, Chan et al. found that cfDNA quantified by a 105-bp leptin fragment was stable for up to 6 h at RT, but increased significantly after 24 h at either RT or 4 ^∘^C [37]. El Messaoudi et al. noted stability within 4 h at both temperatures using BRAF primers targeting a 105-bp sequence but detected significant increases after 6 h at RT. In the same study, the cfDI, calculated as the BRAF-288 bp/BRAF-105 bp ratio, was comparable between samples processed 40 min after collection and those processed after 3 h at RT or 4 ^∘^C, but showed a slight decline after 6 h at RT [11]. Furthermore, Risberg et al. demonstrated that cfDNA quantified using a 65-bp amplicon of RPP30 via digital droplet PCR did not increase significantly within 24 h of delayed processing at RT [38].

Our results highlight that different cfDNA fragments respond variably to storage conditions. LINE1-97 levels remained stable for up to 6 h at both RT and 4 ^∘^C, while LINE1-266 levels were stable for up to 6 h at 4 ^∘^C but increased significantly after just 3 h at RT. Consequently, cfDI values calculated from LINE1-266/97 were higher in plasma obtained from blood subjected to delayed processing. ALU-115 and ALU-247 levels also increased in a time-dependent manner at RT, with greater changes observed for the longer fragment, resulting in elevated cfDI values. These patterns align with the release of gDNA from leukocytes during blood storage [11, 12]. In contrast, at 4 ^∘^C, ALU-115 levels increased significantly only after 6 h, whereas ALU-247 levels remained unchanged for up to 6 h, leading to lower cfDI values at 6 h. Based on these findings, we recommend immediate plasma processing whenever feasible. If delayed processing is unavoidable, blood samples should be stored at 4 ^∘^C for no more than 3 h when analyzing ALU- and LINE1-derived biomarkers.

Data on the impact of long-term plasma storage at −80 ^∘^C on cfDNA concentration and integrity remain limited [39]. Chan et al. found no significant changes in the concentration and cfDI of the leptin fragment after 2 weeks of storage [37], while Sozzi et al. reported substantial cfDNA loss after 4–29 months in certain patient groups, based on quantification of the hTERT fragment [25]. Similarly, El Messaoudi et al. observed that the KRAS fragment remained stable for up to 9 months in a small sample set [11]. In our longitudinal analysis, ALU-derived markers remained stable for up to 12 months. In contrast, LINE1-97 levels declined significantly within the first month, leading to persistent elevation of LINE1-266/97 ratios thereafter. Based on these results, we recommend utilizing ALU-based biomarkers for plasma samples frozen at −80 ^∘^C for up to 12 months, while LINE1-derived biomarkers should be avoided for plasma samples stored for more than one month. These recommendations are particularly relevant for large-scale prospective trials or retrospective analyses involving archived plasma.

This study demonstrated that under storage at 4 ^∘^C, ALU and LINE1 fragments in blood exhibited distinct concentration fluctuations, resulting in differential changes in cfDI. Similarly, plasma storage at −80 ^∘^C led to a significant decrease in the LINE1-97 fragment, while the other three fragments studied remained stable, indicating fragment-specific effects of storage conditions. The mechanisms underlying such fragment-specific stability remain unclear but may relate to sequence-dependent factors, such as differences in chromatin structure, epigenetic modifications, or fragment-end signatures that influence degradation kinetics [7, 31]. These factors may differentially affect the degradation processes of specific cfDNA fragments, leading to the observed variability.

This study has several limitations. First, the relatively small sample size may have limited the statistical power of our analyses and reduced the generalizability of the findings. Larger cohorts are required to validate these preliminary results and confirm that the observed changes in cfDNA concentrations are not attributable to sampling bias. Second, our analysis focused solely on two repetitive elements, ALU and LINE1, representing only a small subset of the repetitive regions within gDNA. Future studies should investigate a broader spectrum of repetitive and gene-specific loci to achieve a more comprehensive understanding of the underlying biological mechanisms. Moreover, research involving more diverse populations with well-characterized clinical and demographic profiles will be essential to determine whether the present findings can be replicated and generalized across different settings and disease conditions.

## Conclusion

This study demonstrates that both blood and plasma storage can alter cfDNA biomarker values measured by qPCR. These effects vary depending on storage duration, temperature, and specific cfDNA fragment analyzed. Therefore, pre-analytical handling, particularly the timing of plasma separation and conditions of plasma storage, should be carefully standardized in cfDNA studies. The choice of storage conditions should be tailored to the specific biomarker targeted, as different cfDNA fragments exhibit distinct stability profiles.

## Supplemental Data

Supplemental data are available at the following link: https://www.bjbms.org/ojs/index.php/bjbms/article/view/13409/4099.

## Data Availability

The datasets used and/or analyzed during the current study are available from the corresponding author on reasonable request.
